# Supplementary material for: Second magnetization peak, anomalous field penetration, and Josephson vortices in KCa2Fe4As4F2 bilayer pnictide superconductor
Source: Sci Rep. 2022 Nov 27;12:20359. doi: 10.1038/s41598-022-24012-z (PMC9701793; doi:10.1038/s41598-022-24012-z)
Supplement: Supplementary file 1 — Supplementary Information. [file 41598_2022_24012_MOESM1_ESM.pdf]

Second magnetization peak, anomalous field penetration, and Josephson vortices in $KCa_2Fe_4As_4F_2$ bilayer pnictide superconductor

P. V. Lopes[1], Shyam Sundar[1,*,†], S. Salem-Sugui, Jr.[1,#], Wenshan Hong[2,3,4], Huiqian Luo[2,5], and L. Ghivelder[1]

[1]Instituto de Fisica, Universidade Federal do Rio de Janeiro, 21941-972 Rio de Janeiro, RJ, Brazil.

[2]Beijing National Laboratory for Condensed Matter Physics, Institute of Physics, Chinese Academy of Sciences, Beijing 100190, China

[3]School of Physical Sciences, University of Chinese Academy of Sciences, Beijing 100190, China

[4]International Center for Quantum Materials, School of Physics, Peking University, Beijing 100871, China

[5]Songshan Lake Materials Laboratory, Dongguan, Guangdong 523808, China

[*]shyam.phy@gmail.com

[#]said@if.ufrj.br

[†]Presently at the School of Physics and Astronomy, University of St. Andrews, KY16 9SS, United Kingdom.

Supplementary Information

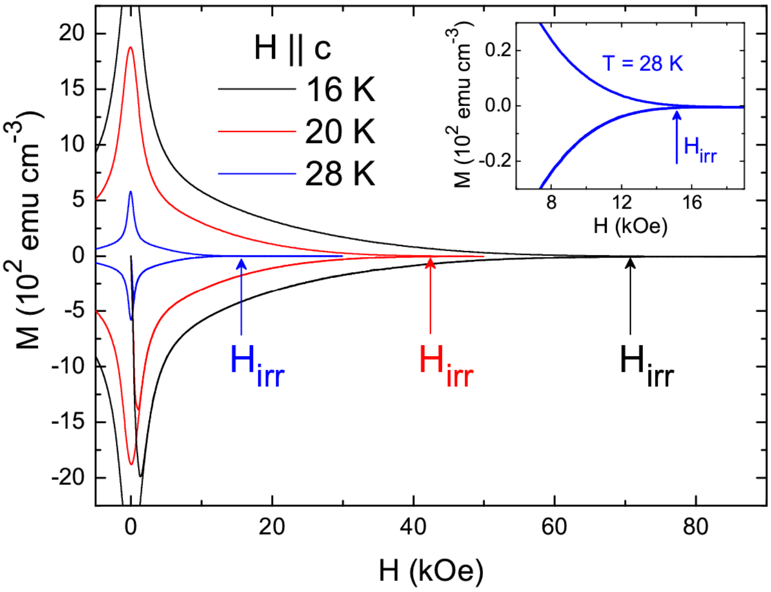

Figure S1: Magnetic field dependence of magnetization measured for H // c-axis. Arrows indicate the irreversibility field H_{irr}, where the field increasing and decreasing branches merge together.

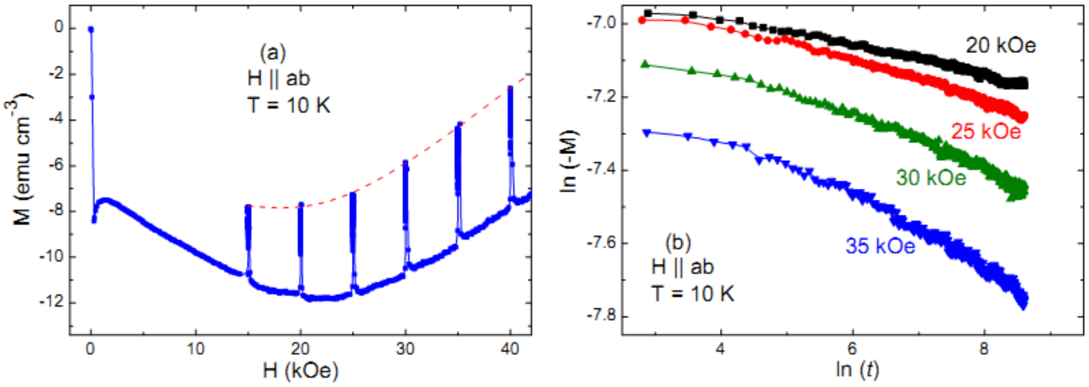

Figure S2: (a) Magnetic relaxation measurements performed on the initial branch of the isothermal M(H), measured at 10 K for H //ab. (b) log-log plot of the magnetic relaxation data shows a non-linear behaviour, which prevented us to obtain relaxation rate for H//ab.

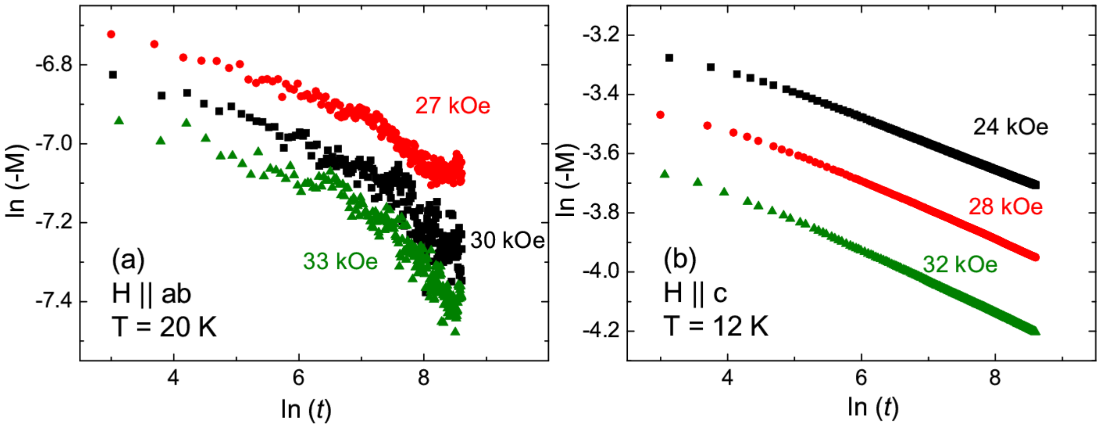

Figure S3: Representative log-log plot of the magnetic relaxation data (a) for H //ab, and (b) for H //c-axis. A non-linear behaviour of ln(-M) vs ln(t) for H //ab, prevented us to obtain relaxation rate.

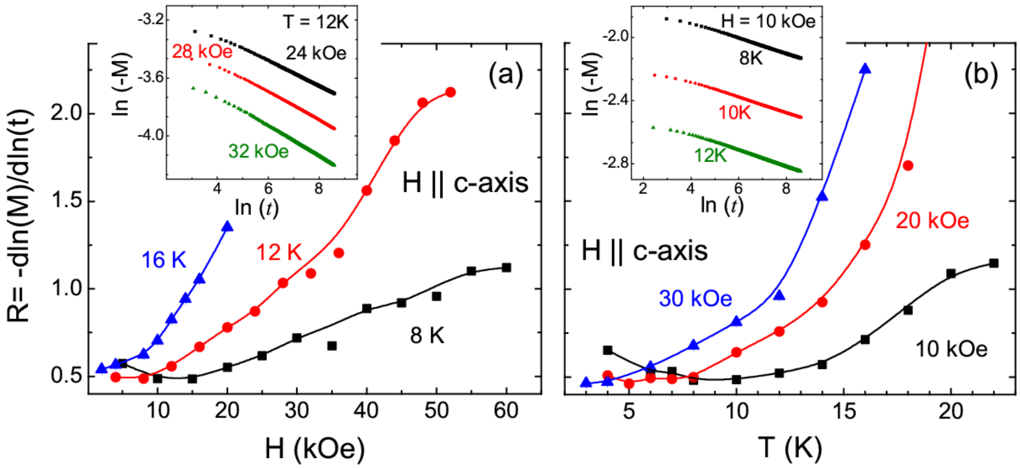

Figure S4: (a) Isothermal magnetic field dependence of relaxation rate at several fixed temperatures for H//c-axis. (b) Isofield temperature variation of relaxation rate at different fixed magnetic fields for H//c-axis. Both insets show the linear behaviour of ln(-M) vs. ln(t) allowed us to obtain the relaxation rate for H//c-axis.

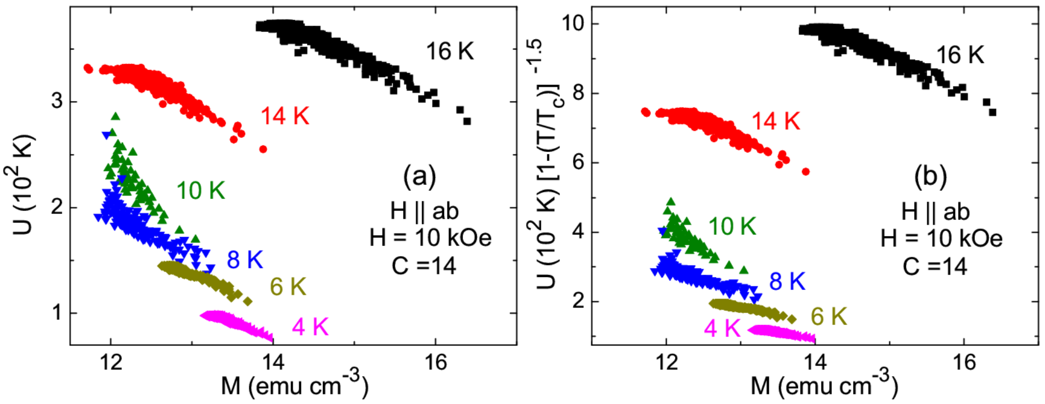

Figure S5: (a) Activation energy as a function of magnetic field U(M), obtained from the magnetic relaxation data measured with H = 10 kOe at various fixed temperatures for H //ab. (b) A failed attempt to scale the U(M) curves using the function $(1\text{-}T/T_c)^{1.5}$.

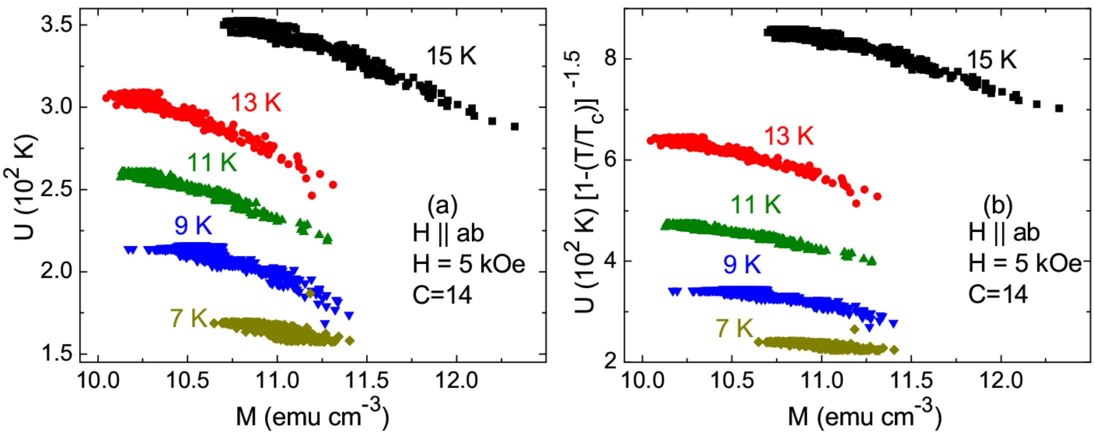

Figure S6: (a) Activation energy as a function of magnetic field U(M), obtained from the magnetic relaxation data measured with H = 5 kOe at various fixed temperatures for H //ab. (b) A failed attempt to scale the U(M) curves using the function $(1\text{-}T/T_c)^{1.5}$.
